# Fine-scale habitat heterogeneity favours the coexistence of supergene-controlled social forms in *Formica selysi*

**DOI:** 10.1186/s12862-020-01742-0

**Published:** 2021-02-14

**Authors:** Sacha Zahnd, Amaranta Fontcuberta, Mesut Koken, Aline Cardinaux, Michel Chapuisat

**Affiliations:** Department of Ecology and Evolution, University of Lausanne, 1015 Lausanne, Switzerland

**Keywords:** Habitat heterogeneity, Competition-colonization trade-off, Supergenes, Spatially varying selection, Social polymorphism, Queen number, Ants, Habitat saturation

## Abstract

**Background:**

Social insects vary widely in social organization, yet the genetical and ecological factors influencing this variation remain poorly known. In particular, whether spatially varying selection influences the maintenance of social polymorphisms in ants has been rarely investigated. To fill this gap, we examined whether fine-scale habitat heterogeneity contributes to the co-existence of alternative forms of social organization within populations. Single-queen colonies (monogyne social form) are generally associated with better colonization abilities, whereas multiple-queen colonies (polygyne social form) are predicted to be better competitors and monopolize saturated habitats. We hypothesize that each social form colonizes and thrives in distinct local habitats, as a result of their alternative dispersal and colony founding strategies. Here, we test this hypothesis in the Alpine silver ant, in which a supergene controls polymorphic social organization.

**Results:**

Monogyne and polygyne colonies predominate in distinct habitats of the same population. The analysis of 59 sampling plots distributed across six habitats revealed that single-queen colonies mostly occupy unconnected habitats that were most likely reached by flight. This includes young habitats isolated by water and old habitats isolated by vegetation. In contrast, multiple-queen colonies were abundant in young, continuous and saturated habitats. Hence, alternative social forms colonize and monopolize distinct niches at a very local scale.

**Conclusions:**

Alternative social forms colonized and monopolized different local habitats, in accordance with differences in colonization and competition abilities. The monogyne social form displays a colonizer phenotype, by efficiently occupying empty habitats, while the polygyne social form exhibits a competitor phenotype, thriving in saturated habitats. The combination of the two phenotypes, coupled with fine-scale habitat heterogeneity, may allow the coexistence of alternative social forms within populations. Overall, these results suggest that spatially varying selection may be one of the mechanisms contributing to the maintenance of genetic polymorphisms in social organization.

## Background

Genetic polymorphisms controlling phenotypic variation within species are widespread in nature, yet in many cases the mechanisms balancing these polymorphisms are unclear and remain debated [1, 2]. Selection varying in space has long been claimed to be an important force maintaining polymorphisms in natural populations [3]. Spatially varying selection occurs when the habitat is heterogenous and alternative alleles have different fitness in distinct habitats [4, 5]. Examples of polymorphisms maintained by spatially varying selection come from a large diversity of taxa. They include resistance to viruses in bacteria [6], shell colour in molluscs [7], dispersal abilities in crustaceans [8] and in insects [9], colour mimetism [10] and resource preference in insects [11].

Social insects vary widely in social organization. Many species have a monogyne social organization, with a single breeding queen per colony. Species with a polygyne social organization, where multiple queens share reproduction in each colony, are also common, particularly in ants [12]. And some species are socially polymorphic, exhibiting both single-queen and multiple-queen colonies, in separate or even within populations [12]. Recent studies in ants uncovered that variation in colony social organization within species is controlled by supergenes in at least three independent lineages [13–17]. This strong genetic basis raises novel questions on the mechanisms maintaining social polymorphisms in time and space. In particular, it is unclear to what extent ecological factors play a role in the maintenance of social polymorphisms.

Spatially varying selection is expected if the monogyne and polygyne social forms differ in their capacity to disperse, reach and settle in different habitats. Striking differences in dispersal and colony founding occur between single-queen and multiple-queen ant species [18–20]. In general, queens of monogyne species disperse on the wing and establish new colonies independently, while queens of polygyne species frequently seek adoption in their natal nests or found new multiple-queen colonies by dispersing on foot with workers (colony budding; [21–23]. Similar differences in dispersal and colony founding strategies have been documented between queens of alternative social forms within polymorphic species [24–28]. These differences in dispersal and colony founding may lead to alternative social forms occupying distinct habitats within populations.

Single-queen colonies (monogyne social form) are predicted to have better colonization abilities, whereas multiple-queen colonies (polygyne social form) are predicted to be better competitors and monopolize saturated habitats. A general view is that ant colonies recruit additional queens in environments with high cost of independent colony founding, for example in saturated habitats with high density of ant colonies or with continuous vegetation (i.e. the habitat saturation hypothesis; [29, 30]. According to this hypothesis, queens of multiple-queen colonies, which can join existing colonies, will be better at colonizing saturated habitats (competitor phenotype). Polygyne colonies may also have other competitive advantages, due to larger colony size, longer colony lifespan and greater genetic diversity leading to better division of labour or disease resistance [31–33]. In contrast, single-queen colonies may preferentially occupy young habitats with available space to found new colonies independently [19, 34, 35]. In addition, due to their higher dispersal abilities, queens of the monogyne social form may be better at colonizing patchy habitats that need to be reached by flight (colonizer phenotype), whereas polygyne colonies may thrive in continuous and connected habitats. Heterogenous mosaic landscapes, comprising a juxtaposition of empty and saturated, connected and discontinuous habitat patches, may thus favour the coexistence of genetically determined social forms varying in social organization, dispersal and mode of colony founding.

Whether the trade-off between competition and colonization favours the coexistence of alternative social forms within species remains untested. Colonization-competition trade-offs are often discussed in the framework of species coexistence, where a species occupies the “colonizer niche” by efficiently colonizing empty habitats, and the other the “competitive niche” by outcompeting the first species locally [36–39]. Two studies, one experimental and the other theoretical, suggest that such trade-offs play a role in the coexistence of ant species with alternative modes of dispersal and colony founding strategies [40, 41]. Here, we investigate if competition and colonization in heterogeneous habitats plays a role in the coexistence of social forms within one ant species.

The Alpine silver ant, *Formica selysi*, has a polymorphic social organization, with both monogyne and polygyne colonies [42]. Most well-sampled populations have both social forms [42, 43], suggesting that the polymorphism is present at a fine geographical scale. This social polymorphism is controlled by a supergene [14, 28]. The supergene is ancient, as it underlies the polymorphic social organization of four other *Formica* species, separated by 20–40 MY of independent evolution [16]. In these species, single-queen and multiple-queen colonies differ in a suite of traits, including dispersal and colony founding strategies [12, 19, 44].

Multiple lines of evidence suggest that *F. selysi* queens originating from monogyne colonies disperse on the wing and found colonies independently, while queens originating from polygyne colonies favour their additional option of staying in their natal colony. Monogyne colonies produce the vast majority of the queens that disperse on the wing to join mating aggregations [31, 45]. Queens originating from polygyne colonies also fly in the field [45] and can found colonies independently in protected laboratory conditions [46], although they are less successful at independent colony founding than queens from monogyne colonies, which have a larger body size [31, 47]. Nestmate queens from polygyne colonies are significantly related on average, which indicates that at least part of them stay within or close to their natal colony [28]. In contrast, monogyne colonies keep only one reproductive queen for their entire lifespan and mature polygyne colonies do not accept queens issued from monogyne colonies [14, 28, 42]. Hence, unlike polygyne queens, monogyne queens do not have the possibility to join an existing nest and are obligate dispersers.

*F. selysi* is a pioneer species that lives in heterogeneous floodplains along rivers in the Alpine region and nests in bare sandy soils [48, 49]. Flood plains are dynamic and rapidly evolving areas. Ecological succession after floods creates a gradient of young to mature ecosystems representing a mosaic of habitats within small geographic areas [50]. Major floods erode soil and eliminate ant nests, creating empty patches available for re-colonization, with varying connectivity due to water bodies. Ant and other arthropod communities typically vary among habitat patches of this mosaic landscape [51, 52]. Alternative forms of ant social organization may differ in their distribution across empty and saturated habitat types found in mosaic floodplains.

Here, we investigate if fine-scale habitat heterogeneity correlates with the distribution of supergene-mediated social forms in *F. selysi*. We search for ecological variables predicting the frequency of single-queen and multiple-queen colonies across patches of habitat, with a focus on the role of habitat age, vegetation (ecological succession) and connectivity (islands vs mainland). Due to the differences in dispersal and life-history between social forms, we expect the monogyne form to monopolize young or unconnected habitats, such as islands or recently flooded areas. In contrast, because of budding and additional queen recruitment, we predict the polygyne social form to monopolize patches of old, saturated and connected habitats.

## Results

*F. selysi* was abundant in the floodplain but had a patchy distribution across the mosaic landscape. Using a systematic search procedure, we detected 354 colonies in 59 plots of 10 × 10 m belonging to six habitat categories (Table 1). The density of *F. selysi* colonies varied greatly among plots (range: 0–22 colonies per plot) and between habitat categories (Table 1; Additional file 1: Table S1). The species occupied all types of young habitat (islands, riverbeds, 8-year old and 16-year old flooded areas), as well as old, open steppe habitat, but was completely absent from old, mature pine forest (Table 1). As expected, the species was socially polymorphic, with 32.6% of the colonies belonging to the monogyne social form, and 67.4% to the polygyne social form (N = 340 colonies; the social organization of 14 colonies could not be determined; Table 1).

**Table 1:** Habitat characteristics and distribution of alternative social forms across habitat types

	Island	Riverbed	Flooded area 8 yo	Flooded area 16 yo	Steppe	Forest
Age since last flooding	Approx. 1 year	Approx. 1 year	8 years	16 years	> 36 years	> 36 years
Number of plots	8	11	15	10	10	5
Number of ant species	2	2	1	1	7	4
Number of colonies per plot (mean ± SD)	5.5 ± 4.1	2.36 ± 1.8	12.66 ± 6.1	8.5 ± 2.1	0.9 ± 1.7	0
Number of monogyne colonies per plot (mean ± SD)	5.38 ± 3.89	0.18 ± 0.60	1.73 ± 2.18	3.2 ± 3.94	0.8 ± 1.48	0 ± 0
Number of polygyne colonies per plot (mean ± SD)	0.13 ± 0.35	2.18 ± 1.94	10.26 ± 6.41	5 ± 4.29	0 ± 0	0 ± 0
Total number of *F. selysi* colonies (number of colonies with undetermined social form)	44 (0)	26 (0)	190 (10)	85 (3)	9 (1)	0 (0)

**Fig. 1:**
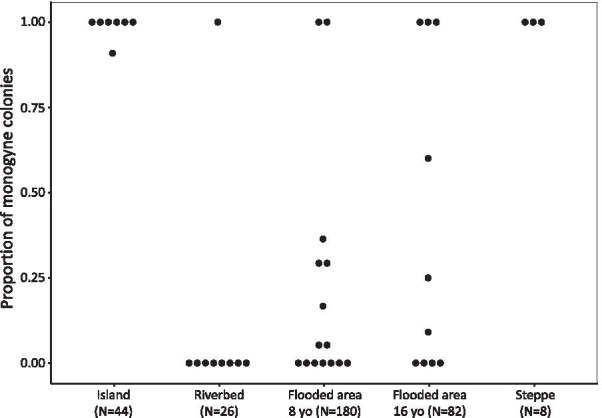
The frequency of social forms varies across habitat types. Monogyne colonies are more frequent on islands and in steppes, while polygyne colonies are more frequent in riverbeds and flooded areas. The proportion of single-queen colonies per plot is indicated, for each habitat category. Dots correspond to plots in which *F. selysi* colonies were sampled, and N indicates the total number of colonies sampled in each habitat category

The proportion of monogyne and polygyne colonies varied greatly between habitat categories (GLM, “habitat category”: df = 42, F = 8.24, p < 0.001; Table 1; Fig. 1). The monogyne social form was by far the most common on islands (97.7% of single-queen colonies on islands, N = 44 colonies) and in steppes (100% of single-queen colonies, N = 8 colonies). In contrast, the polygyne social form was the most common in young mainland habitats, which included riverbeds (92.3% of multiple-queen colonies, N = 26), 8-year old flooded area (85.6% multiple-queen colonies, N = 180) and 16-year old flooded area (61% of multiple-queen colonies, N = 82).

The frequency of alternative social forms was associated with different vegetation types and habitat connectivity levels (mainland versus islands). On mainland, monogyne colonies were more frequent in plots with more vegetation (covered by grass, bushes or trees), whereas polygyne colonies were more frequent in plots with mineral surface (covered by rocks, gravel or sand; Spearman correlation between proportion of mineral surface cover and proportion of polygyne colonies: S = 4420.9, rho = 0.48, p < 0.01). On islands, almost all colonies were monogyne. Island plots were covered by rocks, gravel or sand and were ecologically similar to riverbed plots (Additional file 1: Figure S1). Indeed, islands and riverbeds are annually disturbed by floods and are pioneer, vegetation-poor habitats. Yet islands, which are probably only reachable by flying, were almost exclusively occupied by single-queen colonies, while riverbeds, which can be reached by foot and flight, were almost exclusively occupied by multiple-queen colonies.

**Fig. 2:**
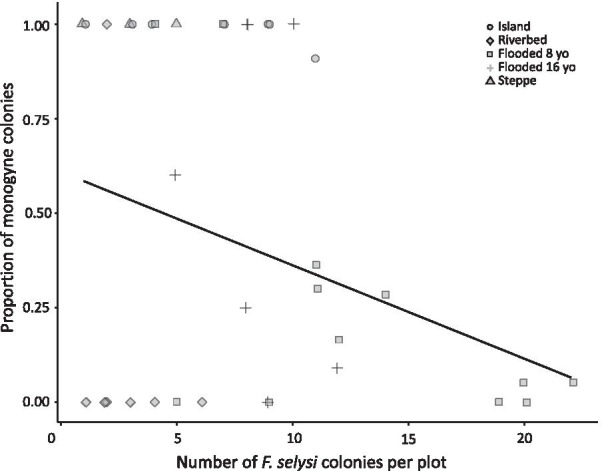
The frequency of social forms varies with colony density. Each point corresponds to a plot, with the symbol indicating its habitat category. The points have been jittered for better visualization. Regression curve represents a simplified GAM model, with the number of monogyne and polygyne colonies as response variable and colony density as fixed factor

The frequency of social forms varied with the density of *F. selysi* colonies. Across all habitat categories, multiple-queen colonies were more frequent in plots with higher colony density (GAMM, “colony density”: df = -0.97, F = 11.60, p < 0.01; Fig. 2; Table 1). The high density of colonies was also associated with low ant species diversity (GAMM, “diversity”: df = -4.19, p = 0.03, Additional file 1: Table S1). In particular, *F. selysi* was the only ant species in mainland young habitats, which are densely populated by multiple-queen colonies (8 yo and 16 yo flooded areas; Additional file 1: Table S1; Fig. 2).

## Discussion

Evolutionary forces maintaining intraspecific polymorphism, such as social organization in ants, are still poorly understood. Alternative forms of social organization are often associated with distinct dispersal and colony-founding strategies. Here, we focus on the interplay between the colonizer and competitor phenotypes of alternative social forms and their success at occupying distinct habitats. We investigated whether and how heterogeneous habitats could favour social polymorphism at a fine geographical scale. We found that in a species with genetically determined social organization*,* the frequency of monogyne and polygyne social forms varied strikingly between habitat types of a mosaic floodplain. Monogyne colonies were more frequent in less populated and more isolated or disconnected habitat patches. In contrast, polygyne colonies were abundant in young, saturated and more connected habitats. This distribution is in line with different dispersal and colony founding strategies, with the monogyne social form exhibiting a colonizer phenotype and the polygyne social form presenting a competitor phenotype. Therefore, our results suggest that social forms play a distinct role in a competition-colonization trade-off, which could promote the coexistence of alternative, genetically determined social forms within populations. To our knowledge, this is the first study to report an association between habitat heterogeneity and the frequency of genetically controlled social forms in the same population.

The polygyne social form monopolized saturated habitats. This is in line with the habitat saturation hypothesis, which predicts that polygyne colonies are abundant in saturated habitats with few available nesting sites [29, 30]. Several studies have found a positive association between the number of queens per colony and proxies of habitat saturation, such as nesting site limitation, invasion gradient and ecological succession [34, 35, 53–56]. Yet, these studies compared social forms in distant locations [54, 57] or assumed that social organization was a plastic trait (e.g. Seppä, Sundström, and Punttila 1995; Ingram 2002; McGlynn 2010). Contrary to what is assumed in the habitat saturation hypothesis, monogyne colonies of *F. selysi* cannot adopt additional queens and mature polygyne colonies never accept young queens of the alternative social origin [28]. Thus, in our system, the abundance of multiple-queen colonies in saturated habitats is due to the success of individuals holding the supergene variant associated with polygyny, rather than to a plastic response of colonies facing changing environmental conditions.

The monogyne social form was more successful than the polygyne one at colonizing the unconnected island habitats. Monogyne colonies represented the large majority of colonies on islands, although we also found one polygyne colony, confirming that polygyne queens have the possibility to disperse by flight. Riverbeds and islands are ecologically similar in vegetation and soil cover, and present abundant and continuous nesting sites, yet they strikingly differ in the proportion of monogyne and polygyne colonies. After severe floods, riverbeds and islands have to be recolonized by females originating from non-flooded areas. Riverbeds are connected to the mainland and may thus be efficiently colonized by workers and queens walking from nearby polygyne colonies (colony budding). By contrast, queens originating from monogyne colonies appear better at reaching and colonizing unconnected habitats.

The oldest habitat, steppes, was occupied exclusively by the monogyne social form. This does not fit the prediction of the habitat saturation hypothesis, whereby polygyne colonies should dominate old, stable habitat with low nest site availability [34, 35, 53, 57]. Steppes are old and mature habitats, with the highest diversity of ants in this floodplain. Suitable nesting sites in sandy patches are isolated amidst dense vegetation and may only be attained by flight. Females from single-queen colonies may thus have an advantage at reaching and colonizing scattered nest sites in steppes. Other traits may also contribute to a better adaptation of the monogyne social form to islands and steppes. Yet, our results suggest that habitat connectivity is a major ecological factor determining the success and distribution of alternative supergene haplotypes that affect both dispersal and social organisation.

Overall, the very unequal distribution of social forms across habitat types suggests that spatially varying selection contributes to the coexistence of alternative supergene haplotypes controlling social organization, dispersal and colony founding strategies. The two social forms appear locally adapted to contrasting habitat types, as in a multi-niche selection framework for dispersal- and competition-related traits [4]. Models predict that a genetic polymorphism for dispersal can be maintained in heterogeneous environments if there is spatial variation in the carrying capacities of patches [59, 60]. Dynamic floodplains show such variation, and selection in spatially heterogeneous environments can thus contribute to maintain the polymorphism within populations. We do not know whether habitat heterogeneity plays a role in the distribution of other socially polymorphic *Formica* species. The maintenance of the polymorphism over 20–40 MY of evolution [16] and across the species ranges [43] likely requires additional mechanisms than spatially varying selection. Yet, our findings highlight the importance of taking spatial distribution and ecological features into account in the study of the evolution and maintenance of supergenes.

## Conclusion

This survey links habitat characteristics to the distribution of supergene-mediated social forms in Alpine silver ants. The mosaic riverine landscape consists in habitat patches that vary in age, vegetation cover and connectivity. The frequency of monogyne and polygyne colonies varies strikingly between habitat types in the same population. Single-queen colonies occupy steppes and islands, while multiple-queen colonies strive in riverbeds and recently flooded areas. Alternative social forms appear to be adapted to colonize and monopolize distinct niches at a very local scale. Overall, these results suggest that habitat heterogeneity, coupled with strong differences in dispersal and colony founding, help to explain the co-occurrence of alternative social forms within populations.

## Methods

### Study site and habitat characteristics

Our study site is a floodplain along the Rhône river, within the Pfyn-Finges Nature Park, in the Valais region, Switzerland (46.311° N, 7.605° E). It comprises a large population of *F. selysi* within a 2 by 1 km area of mosaic habitat [42, 61]*.* Recurrent floods have created a gradient of habitats differing in age and ecological succession stages, from the riverbed to mainland approximately 1 km away from shore. Islands and frequently flooded riverbanks are characterized by a mix of bare sand and gravels, with limited vegetation. Mainland areas that are seldom or never flooded are increasingly covered by vegetation, from steppe to pine forest. Nests of *F. selysi* are typically found in bare sandy soil, usually around or under rocks [48].

We characterized spatial heterogeneity according to habitat age (i.e. time from the last flood), connectivity and vegetation type (Table 1). We determined the date and extent of past floods by looking at orthophotos and high-resolution satellite images from 1980 to 2016 (Swisstopo, aerial photos of 1980, 1998 and 2000; and Google Earth satellite images of 2009, 2013 and 2016). We then classified the area in six contrasted habitat categories (Table 1). Due to the selective sampling strategy, some riverine habitats covered by dense vegetation were not included. Islands are flooded yearly and remain permanently isolated by running water. As they remain under water for several days to weeks, it seems unlikely that ant colonies would survive floods, which is also suggested by the small size of colonies found on islands and riverbeds (pers. obs.). Hence, we assume that island habitat can only be colonized by flying. The riverbed is also flooded yearly, but is otherwise connected to the mainland, and can thus also be reached by foot. Parts of the mainland had been severely flooded in 2000 and in 2008, 16 and eight years before our sampling, respectively. The rest of the mainland, which had not been affected by severe floods over the last 36 years, was divided between steppe dominated by herbaceous vegetation and forest dominated by pine trees.

### Sampling strategy

To investigate if colony social organization varies across habitats, we set up 59 sampling plots of 10 × 10 m, distributed across the six habitat categories (Table 1). The position of each plot within each habitat category was determined randomly using the random points function implemented in the computer program QGIS (version 2.14, [62]). The minimum distance between plots was 25 m. To further characterize the habitat, we measured within each plot the proportion of surface covered by sand, gravel, rock, moss, grass, bushes (less than 50 cm high) or trees. The plots clustered according to the six habitat categories in a Principal Component Analysis (PCA) based on their surface cover, which confirmed that these categories differed in substrate and vegetation cover (Additional file 1: Figure S2). The entire dataset is archived on Dryad, https://doi.org/10.5061/dryad.sj3tx963p.

Colony sampling took place in spring (April–May) and autumn (October), 2016. We located *F. selysi* colonies by following a systematic search procedure based on baiting. Within each plot, we placed 81 baits of tuna and honey on the nodes of a one meter grid square. We waited up to one hour to allow workers of nearby colonies to visit the baits. We then followed the ants back to their nests, and marked all colonies located within the 10 × 10 m plots. Colonies were considered distinct if their entrances were separated by at least one meter [28]. As ant activity depends on weather conditions, baiting was performed only on dry days and when the temperature was above 10 °C.

The social organization of each colony was determined by genotyping three workers per colony at SNPs that are diagnostic for alternative haplotypes of the social supergene (PCR–RFLP assay, developed for the same population, Finges; [14]. The supergene genotype is perfectly associated with the social form of mature colonies: workers from monogyne colonies have exclusively the supergene genotype Sm/Sm, while workers from polygyne colonies have one or two copies of the Sp haplotype [14, 16, 28]. For the 45 plots sampled in the spring, we assessed the presence of other ant species (Additional file 1: Table S1). Workers from other ant species collected on baits were determined by examining their morphology under binocular magnifier and following identification keys [63, 64].

### Statistical analyses

To investigate if the frequency of social forms differed across habitat categories, we ran a binomial Generalized Linear Model (GLM) with the number of monogyne and polygyne colonies per plot as response variable and habitat category as fixed factor. We considered only plots where *F. selysi* was present. We controlled for the sampling period (spring or autumn) by including it as a fixed factor in the model. We adjusted standard errors to account for over-dispersion (quasi-binomial function; [65]).

To investigate the effect of the density of *F. selysi* colonies on the proportion of monogyne and polygyne colonies per plot, we ran a binomial Generalized Additive Mixed Model (GAMM), which handles nonlinear relationships between the response and predictor variables. We included the number of monogyne and polygyne colonies per plot as response variable, the number of *F. selysi* colonies as smoother fixed factor and the habitat category as random factor. We controlled for the sampling period (spring or autumn) by including it as a fixed factor in the model. We adjusted standard errors to account for over-dispersion (quasi-binomial function).

To analyze the effect of ant species diversity on the density of *F. selysi* colonies, we ran a GAMM with Poisson distribution. We included the number of *F. selysi* colonies per plot as response variable, the number of other ant species as smoother predictor, and the habitat category as random factor. All statistical analyses were performed using R 3.5.1 [66]. We used the package “lme4” [67] for GLMs and the package “mgcv” [68] for GAMMs.

## Supplementary Information

**Additional file 1: Table S1.** Ant species abundance across habitat categories. **Figure S1.** Surface cover across plots. Principal component analysis (PCA) of surface cover variables in the 59 sampling plots (proportion of surface covered by sand, gravel, rock, moss, grass, bushes and trees, respectively). The first component (35.1% of the variance) mainly differentiates the substrate (vegetation versus mineral) and the second component (19.6% of the variance) mainly differentiates low vegetation (grass) from high vegetation (bush and trees). Plots in each of the six habitat categories cluster together, which indicates that habitat categories differ in substrate and vegetation. The number of plots is indicated in parentheses. **Figure S2.** Surface cover across plots. Principal component analysis (PCA) of surface cover variables in the 59 sampling plots (proportion of surface covered by sand, gravel, rock, moss, grass, bushes and trees, respectively). The first component (35.1% of the variance) mainly differentiates the substrate (vegetation versus mineral) and the second component (19.6% of the variance) mainly differentiates low vegetation (grass) from high vegetation (bush and trees). Plots in each of the six habitat categories cluster together, which indicates that habitat categories differ in substrate and vegetation. The number of plots is indicated in parentheses.

## Data Availability

All the data are archived on Dryad, https://doi.org/10.5061/dryad.sj3tx963p.

## Abbreviations

GAMM: Generalized additive mixed model
GLM: Generalized linear model
PCR–RFLP: Polymerase chain reaction-restriction fragment length polymorphism
SD: Standard deviation
