# Supplementary material for: Fine-scale habitat heterogeneity favours the coexistence of supergene-controlled social forms in Formica selysi
Source: BMC Ecol Evol. 2021 Feb 14;21:24. doi: 10.1186/s12862-020-01742-0 (PMC7883426; doi:10.1186/s12862-020-01742-0)
Supplement: Supplementary file 1 — Additional file 1: Table S1. Ant species abundance across habitat categories. Figure S1. Surface cover across plots. Principal component analysis (PCA) of surface cover variables in the 59 sampling plots (proportion of surface covered by sand, gravel, rock, moss, grass, bushes and trees, respectively). The first component (35.1% of the variance) mainly differentiates the substrate (vegetation versus mineral) and the second component (19.6% of the variance) mainly differentiates low vegetation (grass) from high vegetation (bush and trees). Plots in each of the six habitat categories cluster together, which indicates that habitat categories differ in substrate and vegetation. The number of plots is indicated in parentheses. Figure S2. Surface cover across plots. Principal component analysis (PCA) of surface cover variables in the 59 sampling plots (proportion of surface covered by sand, gravel, rock, moss, grass, bushes and trees, respectively). The first component (35.1% of the variance) mainly differentiates the substrate (vegetation versus mineral) and the second component (19.6% of the variance) mainly differentiates low vegetation (grass) from high vegetation (bush and trees). Plots in each of the six habitat categories cluster together, which indicates that habitat categories differ in substrate and vegetation. The number of plots is indicated in parentheses. [file 12862_2020_1742_MOESM1_ESM.docx]

## **Additional file**

**Table S1. Ant species abundance across habitat categories**, estimated as the proportion of plots in which a species was detected. Species presence is highlighted in bold (N = number of plots per habitat category). To test if ant species diversity varied across habitat categories, we ran a GLM with a Poisson distribution, with the number of ant species as response variable and the habitat category as predictor. The diversity of ant species differed markedly between habitat categories (GLM, “habitat category”: df = -5, p < 0.01).

|  | **Island**  (N = 3) | **Riverbed** (N = 7) | **Flooded area 8 yo**  (N = 10) | **Flooded area 16 yo** (N = 10) | **Steppe**  (N = 10) | **Forest**  (N = 5) |
| --- | --- | --- | --- | --- | --- | --- |
| *Formica selysi* | **0.67** | **1.0** | **1.0** | **1.0** | **0.3** | 0.0 |
| *Formica cunicularia* | 0.0 | 0.0 | 0.0 | 0.0 | **0.1** | 0.0 |
| *Formica clara* | 0.0 | 0.0 | 0.0 | 0.0 | **0.1** | 0.0 |
| *Myrmica lonae* | 0.0 | 0.0 | 0.0 | 0.0 | 0.0 | **0.2** |
| *Manica rubida* | **0.34** | **0.14** | 0.0 | 0.0 | 0.0 | 0.0 |
| *Plagiolepis vindobonensis* | 0.0 | 0.0 | 0.0 | 0.0 | **0.1** | **0.2** |
| *Tetramorium sp* | 0.0 | 0.0 | 0.0 | 0.0 | **0.1** | 0.0 |
| *Tapinoma erraticum* | 0.0 | 0.0 | 0.0 | 0.0 | **0.1** | **0.2** |
| *Themnothorax parvulus* | 0.0 | 0.0 | 0.0 | 0.0 | **0.1** | **1.0** |

**Figure S1.** Surface cover across plots. Principal component analysis (PCA) of surface cover variables in the 59 sampling plots (proportion of surface covered by sand, gravel, rock, moss, grass, bushes and trees, respectively). The first component (35.1 % of the variance) mainly differentiates the substrate (vegetation versus mineral) and the second component (19.6 % of the variance) mainly differentiates low vegetation (grass) from high vegetation (bush and trees). Plots in each of the six habitat categories cluster together, which indicates that habitat categories differ in substrate and vegetation. The number of plots is indicated in parentheses.

**Figure S2.** Surface cover across plots. Principal component analysis (PCA) of surface cover variables in the 59 sampling plots (proportion of surface covered by sand, gravel, rock, moss, grass, bushes and trees, respectively). The first component (35.1 % of the variance) mainly differentiates the substrate (vegetation versus mineral) and the second component (19.6 % of the variance) mainly differentiates low vegetation (grass) from high vegetation (bush and trees). Plots in each of the six habitat categories cluster together, which indicates that habitat categories differ in substrate and vegetation. The number of plots is indicated in parentheses.
